# On the virome’s hidden diversity: lessons from RHDV

**DOI:** 10.1128/mbio.01971-23

**Published:** 2023-10-19

**Authors:** Ana M. Lopes, Joana Abrantes

**Affiliations:** 1 CIBIO, Centro de Investigação em Biodiversidade e Recursos Genéticos, InBIO Laboratório Associado, Campus de Vairão, Universidade do Porto, Vairão, Portugal; 2 BIOPOLIS Program in Genomics, Biodiversity and Land Planning, CIBIO, Campus de Vairão, Vairão, Portugal; 3 UMIB-Unit for Multidisciplinary Research in Biomedicine, ICBAS-School of Medicine and Biomedical Sciences, University of Porto, Porto, Portugal; 4 ITR-Laboratory for Integrative and Translational Research in Population Health, Porto, Portugal; 5 Departamento de Biologia, Faculdade de Ciências, Universidade do Porto, Porto, Portugal; Albert Einstein College of Medicine, Bronx, New York, USA

**Keywords:** viral evolution, RNA viruses, metatranscriptomics, disease emergence

## Abstract

Emerging infectious diseases are a major challenge to human and animal health. While predicting the emergence of pathogens is complex, the advent of high-throughput sequencing technologies has allowed the rapid identification of unknown microbiology diversity within organisms. Here, we discuss an example of a metatranscriptomics output to decipher viral evolution.

## PERSPECTIVE

The “Meno’s paradox,” or the “Learner’s paradox,” by Plato, debated how a man can learn anything if he does not know what he is searching for. A horde of philosophical arguments over the nature of learning follows the dialog between Meno and Socrates. Research interests often follow anthropogenic needs, and, as such, the knowledge of mammalian viruses has been directed toward those that cause disease in humans or in animals of economic and/or social importance. Yet, healthy organisms are also carriers of viruses, and their role in viral evolution and emergence should not be overlooked. Regrettably, Plato did not live long enough to witness the learning power coming from (viral) metagenomics and metatranscriptomics (collectively, metaviromics [1]). Indeed, metaviromics has revolutionized our understanding of the viral diversity and antiquity and has contributed with numerous descriptions of novel viruses, especially those associated with human and veterinary medicine (e.g., reference 2). The finding of viral communities from unexpected sources, such as saliva and cerebrospinal fluid, is of note (3, 4), with potential applications in therapeutics and surveillance. On the other hand, the high complexity of metaviromics data poses new challenges to this exciting and rapidly expanding field, requiring the constant development of (new) robust computational methods, since the huge amount of sequences produced have no similarity or are only distantly related to those available in public databases. In accord with this, the recent development of a computational tool that searches for the RNA-dependent RNA polymerase, a hallmark gene of RNA viruses, allowed the description of more than 10^5^ novel viruses (5).

In a recent issue of *Cell*, He et al. (6) performed a metatranscriptomic analysis of 18 mammalian species of game animals (in a total of 1,941 specimens) from China to characterize their virome, revealing a great diversity of emerging pathogens. Unsurprisingly, the work focuses on potentially high-risk zoonotic viruses. While we agree that these are the viruses that merit highlighting due to their importance as drivers of disease emergence, we would like to draw attention to what we consider a seamless example of metaviromics as a tool for studying viral evolution. From rabbit samples, He et al. (6) retrieved reads whose assembled contigs were assigned to the lagovirus rabbit hemorrhagic disease virus (RHDV; family *Caliciviridae*, which also includes noroviruses). This RNA virus can cause massive outbreaks and high mortality in European rabbits and, in some instances, in hares, cottontails, and pigmy rabbits, as early as 12–36 hours after the onset of fever (7, 8). Although it may have originated in Europe, the virus was first reported in China in 1984. In the ~40 years of circulation, an array of pathogenic and non-pathogenic forms have been described (9). The hidden diversity in lagoviruses was further reinforced with the emergence, in 2010, of a novel genotype (GI.2), with an unknown origin, which presents a nucleotide similarity of only ~80% to known genotypes and has an orphan structural encoding genomic region (10). Moreover, highly divergent hare lagoviruses have been recovered from healthy animals using metatranscriptomics (11). Notably, the RHDV strains found by He and colleagues are >10% divergent from the closest RHDV strains available in public databases (for comparison, the earliest RHDV strain from 1984 is ~9% divergent from a strain of the same genotype collected 27 years later).

**Fig 1:**
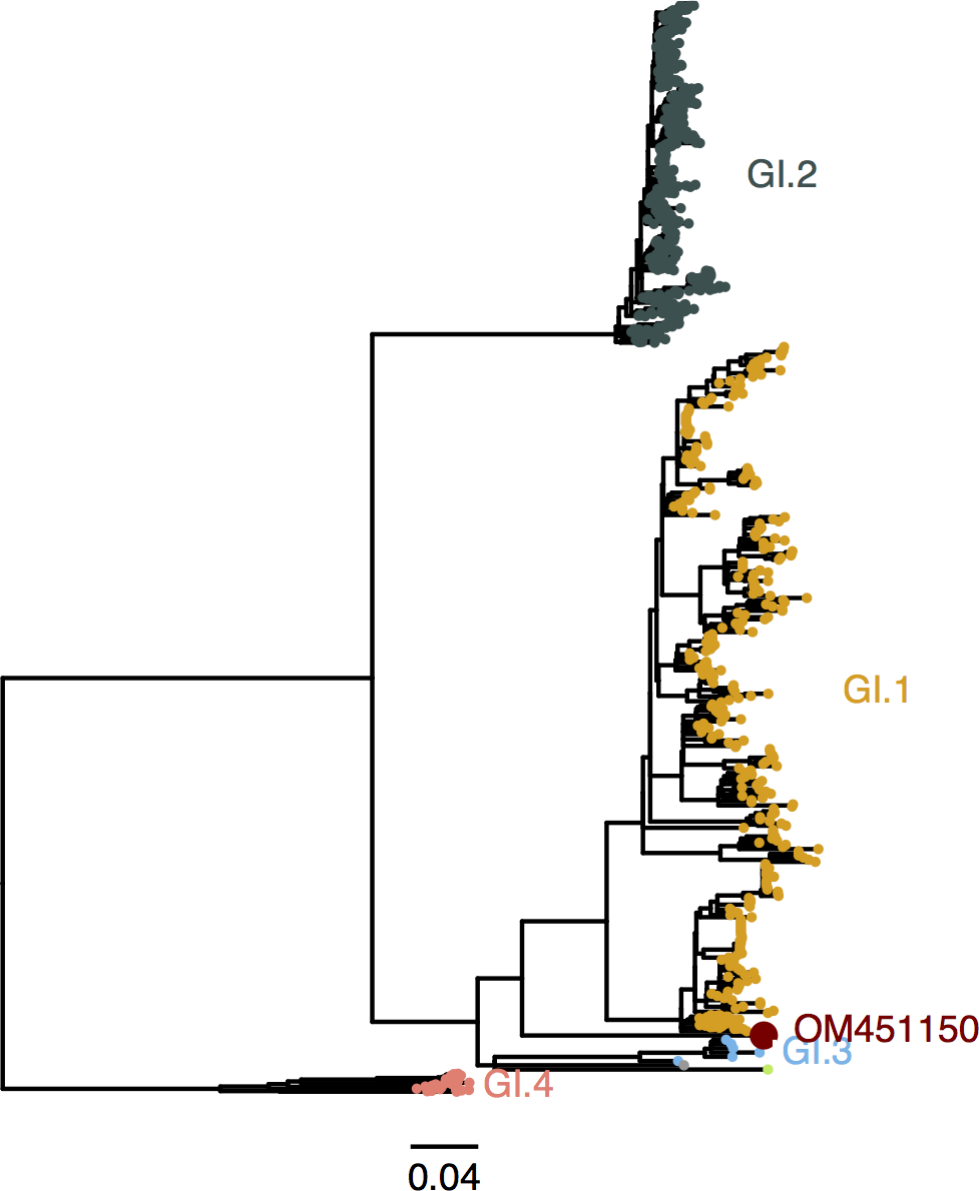
Maximum-likelihood phylogenetic tree of the capsid protein VP60 (*N* = 1,232; 1,740 bp; nucleotide substitution model GTR + G + I). The tree was obtained with MEGA (13). Red: viral genome sequence assembled by He et al. (GenBank accession number: OM451150) (6). Gray: rabbit calicivirus (RCV, GenBank accession number: X96868). Light green: Michigan rabbit calicivirus (MRCV, GenBank accession number: GQ166866). Pathogenic genotypes: GI.1 and GI.2; non-pathogenic genotypes: GI.3 and GI.4. Horizontal branch lengths are drawn to the scale of nucleotide substitutions/site and the tree is midpoint rooted.

The data provided by He et al. (6) might help close a knowledge gap concerning the origin and emergence of lagoviruses. Two non-mutually exclusive hypotheses have been discussed for their emergence: a species jump from sympatric species or the evolution from non-pathogenic forms already circulating in the populations (12). Regarding viral circulation in animals other than the main host(s), as He et al. assert, “[the] capability to carry these viruses, even for a short period of time, may contribute to the virus transmission chains or the emergence of new variants…[which] might in part explain the sometimes sudden and unexpected emergence of new viral variants in humans or domestic animals, as observed in several norovirus outbreaks” (6). Although RHDV has been detected in non-lagomorph mammals, such as vole, shrew, deer, and badger, which might favor the first hypothesis for lagoviruses emergence, their role as (intermediate) hosts is yet to be determined. On the other hand, based on the capsid protein phylogeny (Fig. 1), the strain described by He et al. (6) occupies a basal position to the original GI.1 (pathogenic) genotype. This might be an indicator of its relative ancestry to the strains of the first outbreaks in the early 1980s, thus favoring the second hypothesis for RHDV emergence. The strain, which kept unnoticed until now and was recovered from apparently healthy animals, is indeed more similar to pathogenic RHDV strains, albeit the lack of resolution of the tree in this node. The possible lack of pathogenicity is further supported by the deletion of two amino acids in the capsid protein gene that is only shared with non-pathogenic GI.3 strains that circulate in Europe. The putative European origin of the GI.3 strains is now challenged since they could have been in fact circulating in Asia before being carried to Europe. We hypothesize that these non-pathogenic strains might be the ancestor of GI.1. Coincidentally, the first RHDV outbreaks in 1984 in China were recorded in the Jiangsu province, the same region where the samples from He et al. were collected.

One of the most intriguing biological questions is related to viral origins. Full understanding is often hampered by the traditional action-reaction response, i.e., viral genomes are frequently generated in response to disease outbreaks and not as part of continuous monitoring programs that combine extensive fieldwork and in-depth molecular testing of wildlife, livestock, and farm animals, which are costly and time-consuming. This has been the case with RHDV. Indeed, the first complete RHDV genome was assembled in the early 1990s from a pathogenic strain (14). At the time, this was a remarkable achievement as it represented the first complete calicivirus sequence, but, in the following years, complete genomic sequences accumulated at a slow pace. In addition, and despite mounting evidence, a partial genomic sequence of a non-pathogenic RHDV-related strain became available only in 1996 (15), and complete coding sequences were not produced until 2008 (16). As in many other virus-host relationships, the amount of data for non-pathogenic vs pathogenic lagoviruses is clearly unbalanced: currently, there are ca. 700 complete genomes of pathogenic RHDV strains publicly available with a fairly worldwide distribution, while non-pathogenic strains account for a much smaller fraction with 50 available sequences mostly restricted to Australia. RHDV genomes from China are all from domestic animals, an evidence of the economic-driven study of viral populations. This focus on diseased domestic animals might explain why non-pathogenic Chinese strains had not been detected earlier. Furthermore, sequencing of non-pathogenic strains poses several technical constraints, including a higher sampling effort from healthy animals and the presence of the virus at very low loads. Unfortunately, and as demonstrated by the data generated by He et al. (6), such skewed information might have impaired our understanding regarding lagoviruses evolution, since non-pathogenic strains have been vital in the emergence and evolution of pathogenic strains (e.g., reference 10). Recombination, along with high mutation (and substitution) rates, added extra layers of complexity to the evolutionary history of lagoviruses (e.g., reference 10), emphasizing the need for studying individual host species viromes, including over time, highly relevant.

Viruses have their own evolutionary trajectories that result from an intricate arms race with their hosts, including intermediate hosts. An important take-home lesson from He et al. (6) is that metaviromics greatly extends our knowledge of RNA viruses’ diversity and evolution, and reveals how wildlife is a hotspot of such diversity with a significant role in evolution. A thorough investigation of the huge amount of data produced by metaviromics in such a short time has the potential to provide valuable information, as presented here. The paper from He et al. (6) also reinforces that regular metaviromics surveillance of wildlife, but also zoos and wet markets, could help to promptly detect—and hence, possibly prevent—the emergence and spread of new viral infections. Although there is no current evidence of zoonotic potential in RHDV, rabbits are associated with anthropogenic activities (farming, wild animal hunting, and pet industry) and harbor zoonotic pathogens (17), and thus attention should be directed toward this species.
